# Overview of Monogenic or Mendelian Forms of Hypertension

**DOI:** 10.3389/fped.2019.00263

**Published:** 2019-07-01

**Authors:** Rupesh Raina, Vinod Krishnappa, Abhijit Das, Harshesh Amin, Yeshwanter Radhakrishnan, Nikhil R. Nair, Kirsten Kusumi

**Affiliations:** ^1^Department of Nephrology, Cleveland Clinic Akron General, Akron, OH, United States; ^2^Akron Nephrology Associates, Cleveland Clinic Akron General, Akron, OH, United States; ^3^Department of Pediatric Nephrology, Akron Children's Hospital, Akron, OH, United States; ^4^Department of Medicine, Northeast Ohio Medical University, Rootstown, OH, United States; ^5^Department of Internal Medicine, Carolinas Health Care System Blue Ridge, Morganton, NC, United States; ^6^Department of Internal Medicine, Cleveland Clinic Akron General, Akron, OH, United States; ^7^Department of Biochemistry, Case Western Reserve University, Cleveland, OH, United States

**Keywords:** monogenic hypertension, Liddle syndrome, congenital adrenal hyperplasia, apparent mineralocorticoid excess, Gordon syndrome, familial hyperaldosteronism

## Abstract

Monogenic or Mendelian forms of hypertension are described as a group of conditions characterized by insults to the normal regulation of blood pressure by the kidney and adrenal gland. These alterations stem from single mutations that lead to maladaptive overabsorption of electrolytes with fluid shift into the vasculature, and consequent hypertension. Knowledge of these various conditions is essential in diagnosing pediatric or early-onset adult hypertension as they directly affect treatment strategies. Precise diagnosis with specific treatment regimens aimed at the underlying physiologic derangement can restore normotension and prevent the severe sequelae of chronic hypertension.

## Introduction

Hypertension is one of the most prevalent diseases in the world, affecting an estimated 34% of individuals at or above the age of 20 within the United States alone (1). In contrast, the prevalence of hypertension among individuals aged 12–19 in the United States is 4.11% (2). Treatment of high blood pressure is paramount for improving cardiovascular health and preventing long term morbidity and mortality, per the World Heart Federation (3). The significance of untreated hypertension is emphasized by the severity of end organ damage and extreme systemic sequelae in patients with longstanding disease. The majority of hypertension results from a combination of behavioral and environmental factors; however, various genetic mutations have been identified as distinct causes as well. These genetic forms of hypertension stem from gain- or loss-of-function mutations within the mineralocorticoid, glucocorticoid, or sympathetic pathways (4). The term, monogenic hypertension, is used to describe specific genetic hypertensive disorders which inhibit normal renal and/or adrenal blood pressure regulation. It is especially important to keep these rare conditions in mind when diagnosing hypertension in children, as the younger the child, the more likely that their hypertension is due to a secondary cause. Recent advancements in genetic sequencing methodology have provided further insight into these conditions.

**Figure 1:**
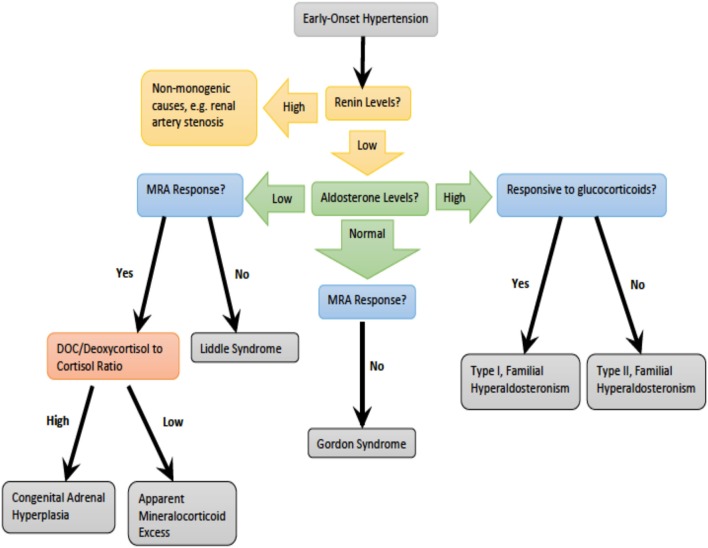
Diagnostic evaluation for monogenic hypertension. DOC, deoxycorticosterone.

Monogenic hypertension drives volume expansion by three distinct mechanisms: (1) excessive sodium ion reabsorption by hyperactive channels, (2) hyperstimulation of mineralocorticoid receptors due to alterations in steroid synthesis, and (3) excess mineralocorticoid synthesis causing volume expansion (5, 6). However, one could conceptualize the latter two mechanisms as indirect means of increasing salt reabsorption, which is the basis of the first mechanism. Monogenic hypertension is further classified based on serum renin and aldosterone levels. Low renin monogenic hypertension conditions can be further categorized by aldosterone level: low, normal, or high (Table 1). Another category is monogenic hypertension due to andrenergic/sympathetic excess. Treatment must be specific to each syndrome's unique derangements within the mineralocorticoid, glucocorticoid, or sympathetic pathways. However, as with all types of hypertension, treatment is aimed at the amelioration of cardiovascular morbidity and mortality associated with long standing high blood pressure. The various types of monogenic hypertension with specific evaluation and treatment considerations will be reviewed in this article. Table 2 provides an overview of the causes of monogenic hypertension and their Online Mendelian Inheritance in Man (OMIM) numbers (7). Figure 1 depicts the diagnostic evaluation for monogenic hypertension.

**Table 1:** Basic classification scheme for causes of monogenic hypertension.

Low renin level	Low aldosterone levels	Liddle syndrome Congenital adrenal hyperplasia Apparent mineralocorticoid excess Gellers syndrome
	Normal aldosterone levels	Gordon syndrome (pseudohypoaldosteronism type II)
	High aldosterone levels	Familial hyperaldosteronism type I (glucocorticoid-remediable aldosteronism) Familial hyperaldosteronism type II Familial hyperaldosteronism type III Familial hyperaldosteronism type IV
Adrenergic/sympathetic excess	High metanephrine and normetanephrine levels	Familial pheochromocytoma
Vascular smooth muscle proliferation		Hypertension and brachydactyly syndrome

**Table 2:** Overview of the causes of monogenic hypertension and their OMIM genotype and phenotype numbers.

**Condition**	**Mode of inheritance**	**OMIM phenotype number(s)**	**OMIM genotype numbers(s)**	**Cytogenetic loci**	**Pathophysiology**	**Management**
Liddle syndrome	Autosomal dominant	177200	600760 (SCNN1B) 600761 (SCNN1G)	16p12.2	Hyperactive ENaC reabsorbs sodium at elevated levels, resulting in volume expansion and hypertension	Patients present with early onset HTN with hypokalemia non-responsive to conventional therapy. Genetic testing confirms the diagnosis. Use ENaC inhibitory agents: amiloride, triamterene.
Congenital adrenal hyperplasia	Autosomal recessive	202010 (type IV)	610613 (CYP11B1)	8q24.3	Defects in steroid synthesis cause buildup of intermediate metabolites with MR activity	Patients present with HTN at very young ages along with atypical sexual development . Glucocorticoid supplementation to suppress ACTH expression treats HTN; potentially add MR antagonists for better control. Therapy should also be individualized to address aspects of sexual dysfunction.
	Autosomal recessive	202110 (type V)	609300 (CYP17A1)	10q24.32		
Syndrome of apparent mineralocorticoid excess	Autosomal recessive	218030	614232 (HSD11B2)	16q22.1	HSD11B2 deficiency allows excess cortisol stimulation at the MR	Therapy uses MR antagonists to alleviate overactivity and may call for ACTH suppression with excess cortisol
Geller syndrome	Autosomal dominant	605115	600983 (NR3C2)	4q31.23	Genetic mutations in the MR alter its structure and binding affinities, allowing atypical stimulation by other steroids, especially progesterone	Presents by early adult life; most critical in pregnant women. Management would be with delivery of the child and subsequent monitoring. Spironolactone is to be avoided.
Gordon syndrome (pseudohypoaldosteronism type II)	Autosomal dominant	145260 (type IIA)	Unspecified	1q31-1q42	Mutations in regulatory proteins for the NCC channel allow for unchecked activity, causing subsequent electrolyte and fluid overabsorption	Thiazide diuretic therapy directly treats NCC hyperactivity.
	Autosomal dominant	614491 (type IIB)	601844 (WNK4)	17q21.2		
	Autosomal dominant	614492 (type IIC)	605232 (WNK1)	12p13.33		
	Autosomal recessive or dominant	614495 (type IID)	605775 (KLHL3)	5q31.2		
	Autosomal dominant	614496 (type IIE)	603136 (CUL3)	2q36.2		
Familial hyperaldosteronism type I (glucocorticoid-remediable aldosteronism)	Autosomal dominant	103900	610613 (CYP11B1)	8q24.3	Unequal crossing over between the CYP11B1 and CYP11B2 genes generates a chimeric product that is ACTH-sensitive and produces aldosterone	Treatment with glucocorticoids to reduce ACTH secretion, supplemented with MR antagonists if necessary. Patients should be screened regularly for HTN-induced cerebrovascular sequelae
Familial hyperaldosteronism type II	Autosomal dominant	605635	600570 (CLCN2)	3q27.1	Hyperplasia or benign neoplasia within the adrenal cortex results in excess aldosterone production	Medical management with MR antagonists with potential surgical resection
Familial hyperaldosteronism type III	Autosomal dominant	613677	600735 (KCNJ5)	11q24.3	Gain-of-function mutations in potassium channels allow adrenal cortical cells to depolarize and subsequently activate aldosterone synthase	Medical management with MR antagonists with potential surgical resection
Familial hyperaldosteronism type IV	Autosomal dominant	617027	607904 (CACNA1H)	16p13.3	Gain-of-function mutations in calcium channels delay inactivation of cells, allowing enhancing aldosterone synthase activity	Medical management with MR antagonists with potential surgical resection
Familial pheochromocytoma	Autosomal dominant	171300	605995 (KIF1B)	1p36.22	Neoplasia of the adrenal medulla generates heightened levels of norepinephrine and epinephrine	Medical management with catecholamine antagonists and other antihypertensives prior to surgical resection. Continuous monitoring and genetic testing may prove helpful with syndromic causes
			185470 (SDHB)	1p36.13		
			613403 (TMEM127)	2q11.2		
			608537 (VHL)	3p25.3		
			600837 (GDNF)	5p13.2		
			164761 (RET)	10q11.21		
			602690 (SDHD)	11q23.1		
			154950 (MAX)	14q23.3		
Hypertension and Brachydactyly Syndrome	Autosomal dominant	112410	123805 (PDE3A)	12p12.2	Gain-of-function mutations generate increased cAMP levels causing enhanced vascular smooth muscle proliferation, accompanied by brachydactyly due to dysfunctional chondrogenesis	High concentration milrinone therapy with possible benefits from phosphodiesterase inhibitors to increase cGMP levels

*Gene names are in parentheses next to the genotype number, where applicable. HTN, hypertension; ENaC, epithelial sodium channel; ACTH, adrenocorticotropic hormone; MR, mineralocorticoid receptor; NCC, sodium chloride cotransporter*.

## Low Aldosterone Monogenic Hypertension

**Case 1**: A 10-year-old boy presented to the clinic with skeletal muscle weakness, paresthesia, nausea, and vomiting, all starting ~5–6 months ago. He had no significant past medical history and was previously healthy. His mother had been diagnosed with hypertension at the age of 20 years, but no other significant family history was noted. The patient was not currently on any medications and had no known drug allergies. On examination, the child was mildly lethargic. Blood pressure measured 164/92 mmHg in the arms and 183/112 mmHg in the legs. The rest of the general physical examination was unremarkable. The patient's height and weight were at the 50th and 62nd percentile, respectively, based on the WHO growth chart. Laboratory work up included complete blood count, electrolytes, thyroid profile, liver profile, urinalysis (UA), and abdominal imaging was obtained. The patient's electrolytes were significant for hypokalemia with serum potassium levels of 2.1 mEq/L (normal 3.5–5.0 mEq/L) as well as a mild metabolic alkalosis with serum bicarbonate of 31 mmol/L (normal 22–29 mmol/L). The remaining labs and imaging studies were unremarkable. The patient was started on spironolactone for the elevated blood pressure and potassium supplements for hypokalemia and was asked to return for a follow up visit in 2 weeks.

After 2 weeks, the patient returned to the clinic with no change in symptoms. Lab values remained the same despite appropriate medication adherence. Plasma renin, serum aldosterone, 11-beta-hydroxylase, and 17-alpha-hydroxylase were ordered. Lab values revealed suppressed plasma renin <1.0 ng/mL/h (normal 2.8–39.9 ng/mL/h) as well as low aldosterone level 8.2 ng/mL/h (normal >20 mg/mL/h). The plasma aldosterone to plasma renin ratio was greater than 30 ng/dL. The child was diagnosed with Liddle syndrome and was started on amiloride. At the next follow up visit, the patient's prior symptoms resolved, blood pressure had returned to the normal range based on age, sex, and height; and the hypokalemia had resolved.

## Liddle Syndrome

**Figure 2:**
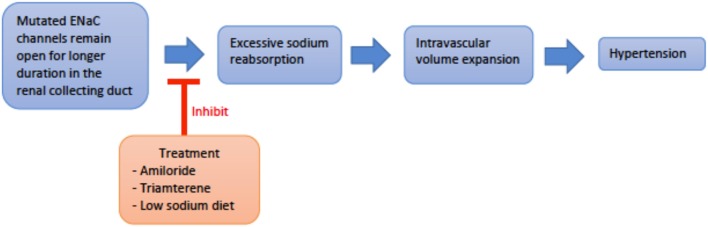
An overview schematic of Liddle svndrome pathophysiology and treatment. ENaC, epithelial sodium channel.

*Genetics and pathogenesis*: Liddle Syndrome is caused by an autosomal dominant (AD), gain-of-function mutation of the epithelial sodium channel (ENaC) present in the collecting duct. The ENaC beta and gamma subunits, SCNN1B and SCNN1G, respectively, are most commonly affected (4, 8). Mutation of these subunits disrupts expression of proline-rich regions of the cytoplasmic carboxyl terminus and results in loss of regulatory binding sites for Nedd4-2, a ubiquitin ligase necessary for the breakdown of ENaC (8–10). Thus, ENaC remains constitutively active with increased Na^+^ reabsorption and subsequent intravascular volume expansion culminating in hypertension (Figure 2) (8, 11, 12). Liddle Syndrome-associated Na^+^ reabsorption is independent of aldosterone, and a hallmark of the disease is the suppression of renin and aldosterone. Hypokalemia ensues as excess Na^+^ is continually absorbed with extrusion of K^+^ via the Na/K-ATPase pump (8, 13). Mild metabolic alkalosis is also seen, as the increased Na^+^ reabsorption increases the net negativity of the lumen, causing an increase in H^+^ extrusion via the renal outer medullary potassium (ROMK) channel and H^+^-ATPase pump located on alpha-intercalated cells (10).

*Epidemiology*: Liddle's is rare with only 30 patients having been formally diagnosed since 2008; however, many nephrologists perceive that it is an under recognized cause of hypertension (14). Liddle's has an estimated prevalence of 1.5% with genetic testing in a Chinese population and was found to have a 6% prevalence in hypertensive patients among a study of US veterans in Louisiana (15, 16). Age of onset for hypertension is typically young, between late childhood and adolescence (17). Patients lacking a family history have also been noted, indicating that LS should always remain in the differential for early-onset hypertension irrespective of the family history (8).

*Work Up*: A strong family history of hypertension, suppressed renin/aldosterone levels and response to ENaC antagonism is highly suggestive, but a definitive diagnosis requires genetic testing (16). Random aldosterone/renin ratio can be used as a screening test, and a ratio >30 excludes the diagnosis (when expressed in ng/dL for aldosterone and ng/dL/h for renin) (15). Renin is suppressed by both elevated sodium levels and volume expansion; aldosterone is often less suppressed, resulting in the increased aldosterone/renin ratio. Liddle syndrome is alternatively known as pseudohyperaldosteronism due to its similarity to hyperaldosteronism with hypertension, hypokalemia, and metabolic alkalosis (18).

*Treatment*: Treatment consists of direct ENaC inhibition with potassium-sparing diuretics such as amiloride or triamterene. Spironolactone is not useful as the genetically altered ENaC is independent of mineralocorticoid regulation (8). A low sodium diet is also important in managing hypertension and counteracting the altered ENaC physiology (19).

**Case 2:** A 10-year-old female presented with hirsutism, particularly on her face as well as the pubic area. She first noticed the hair development 2 months ago. Her past medical history is significant for menarche at the age of 8 with irregular menstrual periods accompanied by breast development. Her maternal aunt also went through puberty around the age of 7–8. The patient was not on any medications at the time of office visit. On examination, the patient was alert with no signs of distress. Her blood pressure measured 168 /104 mmHg on manual auscultation. She is currently Tanner stage II, though her clitoral folds were noted to be enlarged. She is at the 80 and 90th percentiles for height and weight, respectively. Pertinent labs including a complete metabolic panel, adrenal profile, and thyroid profile were obtained. Lab results showed hypokalemia as well as elevated levels of multiple hormone precursors and androgens [serum potassium 2.1 mEq/L (normal 3.5–5.0 mEq/L), serum deoxycorticosterone 21 ng/dL (normal 2–13 ng/dL), serum dehydroepiandrostenedione sulfate (DHEAS) 392 μg/dL (normal <160 μg/dL), serum testosterone, 82 ng/dL (normal 0–30 ng/dL)]. Based on presentation and laboratory findings, the patient was diagnosed with congenital adrenal hyperplasia due to 11β-hydroxylase deficiency. She was started on hydrocortisone with subsequent resolution of her hypertension and hypokalemia.

## Congenital Adrenal Hyperplasia

Though 21α-hydroxylase deficiency is more common, 11β-hydroxylase deficiency (11OHD or CAH type IV) and 17α-hydroxylase deficiency (17OHD or CAH type V), are two subtypes of CAH known to cause monogenic hypertension (13). The respective enzymes regulate different steps in steroid synthesis, but 11OHD and 17OHD deficiency both cause elevated deoxycortisol and deoxycorticosterone levels. These intermediates have activity at the mineralocorticoid receptors. Unchecked mineralocorticoid activity leads to hypertension and hypokalemia. Both types are inherited in an autosomal recessive (AR) fashion, stemming from inactivating mutations that prevent expression of the respective enzymes (20).

### 11β-Hydroxylase Deficiency

**Figure 3:**
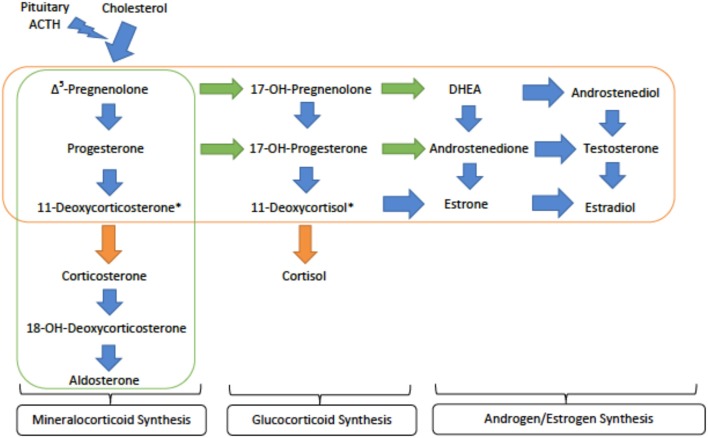
An overview of the steroid synthesis pathways and the enzyme deficiences that lead to congenital adrenal hyperplasia (CAH) types IV and V. The orange arrows show the activity of 11β-hydroxylase (deficient 1 in type IV) while the orange rectangle surrounds the steroid hormones still produced in CAH type IV. The green arrows denote the activity of 17α-hydroxylase (deficient in type V) while the green rectangle encircles those hormones produced in CAH type V. ACTH - adrenocorticotropic hormone; DHEA - dihydroepiandrosterone. ^*^These metabolites are active at the mineralocorticoid receptor and are important in generating hypertension.

*Genetics and Pathophysiology:* CAH type IV, or loss of 11β-hydroxylase, prevents conversion of deoxycortisone and deoxycortisol into corticosterone and cortisol, respectively. Both deoxycortisone and deoxycortisol have weak activity at the mineralocorticoid receptor, but their accumulation in CAH type IV leads to significant mineralocorticoid activity and subsequent hypertension. As the conversion to glucocorticoids, androgens and estrogens are preserved, patients present with high levels of deoxycorticosterone, deoxycoritsol and androgens, mainly androstenedione and dehydroepiandrosterone (DHEA) (Figure 3). This is a key factor in laboratory diagnosis after ACTH (adrenocorticotropic hormone) stimuation, as high levels of the androgens coupled with HTN prove helpful in differentiating 11OHD from other causes of CAH and monogenic hypertension (21).

*Epidemiology:* 11OHD is considered to be a rare condition that accounts for 5–8% of CAH cases (22). However, it has been seen with relatively high frequency among certain populations and accounts for 15% of CAH cases in Muslim and Jewish Middle Eastern populations (23). Detection of this disorder can be done by testing for 11β-hydroxylase activity or measuring tetrahydro-11-deoxycortisol in amniotic fluid or urine.

*Workup:* Like other CAH subtypes, 11OHD manifests with disorders of sexual development. Patients can potentially be identified at birth based on physical findings. Due to androgen accumulation, female infants undergo virilization, presenting with ambiguous genitalia at birth with enlarged clitoral folds (4). Androgen excess can also cause precocious puberty in both males and females. Regardless of variations in sexual development, patients present with hypertension at very young ages. Diagnosis relies on measuring levels of the adrenal steroid hormones to identify enzyme activity and defects. Further genetic testing for the CYP11B1 gene (cytogenetic locus 8q24.3) offers a definitive understanding of disease pathophysiology, guiding treatment (21).

*Treatment:* Treatment consists of glucocorticoid doses sufficient to decrease ACTH secretion, inhibiting stimulation of steroid synthesis and accumulation of mineralocorticoid receptor agonists. Spironolactone, amiloride, and calcium channel blockers may be further used to treat hypertension. Further considerations include individualized therapy to accommodate sexual development in patients and genital malformations in females may need surgical correction (24).

### 17α-Hydroxylase Deficiency

CAH type V, also known as P450C17α deficiency, manifests clinically as hypogonadism, hypokalemia, and hypertension. The gene, CYP17A1, located at cytogenetic locus 10q24.32 encodes 17α-hydroxylase with two key steroidogenic functionalities: 17α-hydroxylase and 17,20-lyase activity (21). Its 17α-hydroxylase function generates 17α-hydroxypregnenolone and 17α-hydroxyprogesterone, which can be converted to cortisol. These products of the 17α-hydroxylase are also substrates for the 17,20-lyase, which generates DHEA and androstenedione to serve as precursors for androgen and estrogen synthesis. This rare disorder thus blocks the production of cortisol as its direct precursors are not synthesized, shunting steroid production toward the mineralocorticoids (Figure 3). Since the blockage is so early in the pathway, there is very little production of sex hormones (23). Male patients have ambiguous external genitalia and may even exhibit a female phenotype, while females present with primary amenorrhea and delayed sexual development (6).

*Workup:* Steroid analysis upon ACTH-stimulation would lead to an appropriate diagnosis, showing atypically elevated levels of pregnenolone and progesterone relative to 17α-pregnenolone and 17α-progesterone, respectively.

*Treatment:* Treatment for these patients would be the same as type IV regarding hypertension with the addition of sex hormone therapy (6).

**Case 3:** A 10-month-old infant came to the clinic with severe hypertension. The patient had no significant past medical history. Lab results as well as imaging reveal severe hypokalemia, mild left ventricular hypertrophy (LVH), and nephrocalcinosis on renal ultrasound. Further metabolic lab studies showed low aldosterone and renin levels. A 24-h urine tetrahydrocortisol (THF) + allo-tetrahydrocortisol (5α-THF) to tetrahydrocortisone (THE) was collected when prior labs detected normal deoxycorticosterone, corticosterone, 18-hydroxydeoxycorticosterone, and 18-hydroxycortisol levels. The 24-h urine sample of THF + 5αTHF confirmed the diagnosis of syndrome of apparent mineralocorticoid excess (AME). The patient was started on amiloride with potassium supplements. A follow up visit in 2 weeks showed normalization of blood pressure as well as normalized potassium.

## Apparent Mineralocorticoid Excess

**Figure 4:**
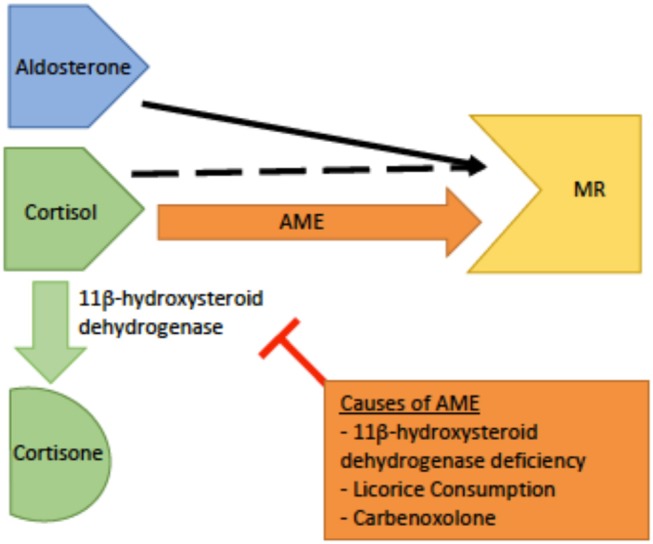
A schematic of the pathophysiology of apparent mineralocorticoid excess (AME). The dashed black arrow indicates normal binding of cortisol to the mineralocorticoid receptor (MR) with conversion to cortisone by 11β-dehydrogenase.The orange arrow indicates AME. In AME, deficiency or inhibition of 11β -dehydrogenase leads to increased levels of cortisol increased cortisol-MR binding, and subsequent hyperactivity of the MR.

*Genetics and pathophysiology*: The syndrome of apparent mineralocorticoid excess (AME) is an autosomal recessive disease caused by an inactivating mutation of 11β-hydroxysteroid dehydrogenase type 2 (HSD11B2). The cytogenetic location is on chromosome 16q22.1 (4). The mineralocorticoid receptor's prototypical ligand is aldosterone, but cortisol is also able to bind and activate the receptor. Thus, the primary function of HSD11B2 is to prevent cortisol from binding to the mineralocorticoid receptor by catalyzing its metabolism to cortisone which does not have mineralocorticoid activity (6, 23). As cortisol is expressed at much higher physiologic levels than aldosterone, HSD11B2 function is critical to maintaining proper control over mineralicorticoid receptor activation. With this mutation, cortisol is not metabolized and is able to bind to the mineralocorticoid receptor, causing clinical features similar to pseudohyperaldosteronism (Figure 4). However, in this disorder the defect is at the level of the receptor itself rather than down stream at the ENaC channel. However, due to this similarlity both groups of patients typically present with hypokalemia, hypertension, and metabolic alkalosis (25).

*Epidemiology*: AME has been observed in many ethnic groups, including Caucasians, African Americans, Asians, and American Indians. This disease presents in early childhood. Severe phenotypes can present with low birth weight and failure to thrive, and if left untreated, these patients have high mortality rates (13).

*Workup*: The diagnosis is based on laboratory findings of hypokalemia, metabolic alkalosis, hyporeninemia, hypoaldosteronemia, and an elevated urinary cortisol to cortisone ratio by THF + 5αTHF to THE in a 24-h urine collection (26). It is important to rule out chronic excess licorice or carbenoxolone ingestion; as these cause similar symptoms due to inhibition of the same enzyme as in AME, but typically resolve once the offending agent is removed (24).

*Treatment*: Treatment consists of mineralocorticoid receptor antagonists, such as spironolactone or eplerenone, along with potential potassium supplements and dietary sodium restriction (6, 24). Elevated cortisol levels may further warrant glucorticoid therapy to reduce ACTH-stimulated cortisol production and subsequent mineralocorticoid receptor stimulation (19, 27). Renal transplantation may be curative in some cases (28).

**Case 4**: A 17-year-old with a past medical history of hypertension came in for her routine checkup for her 34-week-old fetus. She had a family history of elevated blood pressure on her father's side. She complained of mild headaches for the past couple of weeks. On exam, her blood pressure was severely elevated. Laboratory testing including basic metabolic panel, thyroid function tests, and serum cortisol came back normal. Brain CT was normal. Her hypertension was attributed to pre-eclampsia and the patient was admitted for emergent C-section. After delivery, the patient's blood pressure was still mildly elevated and she received a dose of spironolactone. Two hours after taking the medication, her blood pressure worsened instead of improving. The patient was given other antihypertensives and her blood pressures improved. Genetic testing revealed a mutation of the mineralocorticoid receptor gene to confirm the diagnosis of Geller Syndrome.

## Geller Syndrome

Geller syndrome, also referred to as constitutive activation of the mineralocorticoid receptor, results from a gain-of-function mutation on chromosome 4q31. Geller syndrome has an autosomal dominant inheritance pattern (17). This mutation within the mineralocorticoid receptor causes it to remain constitutively active due to changes to receptor sites that result in altered specificities to the steroid hormones. Steroid hormones, including progesterone, subsequently act as agonists of the mineralocorticoid receptor, as opposed to their normal antagonism (5).

The onset of hypertension starts before the age of 20 (17). It is essential to recognize this disease, as pregnancy can severely exacerbate the hypertension due to elevated progesterone levels. This causes activation of the mineralocorticoid receptors, though both aldosterone and renin are suppressed. Potassium levels are surprisingly normal in these patients. A clear diagnosis can be done by genetic testing for gene mutations in the mineralocorticoid receptor (17). Treatment is unlike many other causes of monogenic hypertension, as spironolactone is contraindicated and will worsen the disease. The mainstay for treatment during pregnancy is to deliver the fetus. Optimal management of non-pregnant females and males is not well-defined.

## Normal Aldosterone Monogenic Hypertension

**Case 5**: A 9-year-old female was brought into the clinic by her mother for new onset dizziness and muscle weakness. The patient's height and weight were normal based on the WHO growth chart. Laboratory testing including chemistries were notable for severe hyperkalemia and hypocalcemia. All other lab values including PTH were normal. On exam her blood pressure was very elevated at 145/90 prompting renin and aldosterone testing. Renin levels were below normal and aldosterone was at the upper limits of normal. The patient was diagnosed with Gordon syndrome and she was started on a low dose thiazide.

### Pseudohypoaldosteronism Type II (Gordon Syndrome)

**Figure 5:**
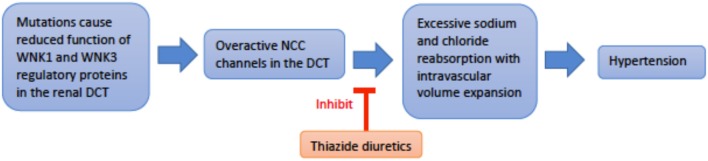
An overview schematic of Gordon Syndrome pathophysiology and treatment. NCC is the sodium chloride co-transporter; DCT is the distal convoluted tubule.

*Genetics and pathophysiology*: Pseudohypoaldosteronism type II (PHAII), also referred to as Gordon syndrome, is autosomal dominant and affects the WNK serine-threonine kinase family (WNK1 and WNK4) (29). PHAII type B is due to a loss-of-function mutation of the WNK4 gene on chromosome 17q21.2, while PHAII type C results from gain-of-function mutation of the WNK1 gene on chromosome 12p12.3 (30). The most common clinical features seen are hyperkalemia, hyperchloremic metabolic acidosis, and normal-to-elevated levels of aldosterone. Mutation of the WNK genes leads to failure of endocytosis of the Na^+^-Cl^−^ co-transporter (NCC). This loss of inhibitory regulation of NCC in the distal convoluted tubule causes hyperchloremic metabolic acidosis. More specifically, WNK4 activity directly inhibits NCC activity by reducing its expression on the extracellular membrane. In contrast, WNK1 inhibits WNK4 to reduce inhibition of NCC expression on the apical membrane. Thus, a loss-of-function in WNK4 or gain-of-function in WNK1 both generate a PHAII phenotype. The resulting increase in sodium reabsorption in the distal convoluted tubule inhibits normal sodium reabsorption downstream by ENaC (Figure 5). Without ENaC sodium reabsorption, ROMK potassium excretion as well as H-K exchange are both suppressed, driving the hyperkalemia and acidosis associated with Gordon's (13). Hypercalciuria is also found in these patients, and specifically hypercalciuria in the absence of elevated parathyroid hormone or serum calcium. The mechanism of urine calcium wasting is not completely understood, though multiple mechanisms have been postulated. WNK4 is known to modulate the activity of the transient receptor potential V5 channel (TRPV5), also located in the distal convoluted tubule. Decreased WNK4 function may thus decrease the normal inflow of calcium ions through TRPV5 in the DCT. Increased plasma sodium levels in PHAII may also inhibit the function of the Na^+^/Ca^2+^ exchanger in the PCT. Ultimately, reduced calcium reabsorption places these patients at risk for urolithiasis and osteoporosis (31, 32).

Two additional genes, KLHL3 and CUL3, located at chromosome 5q31.2 and 2q36.2, respectively, have been identified as aiding WNK4 function; KLHL 3 mutations are implicated in PHAII type D, while CUL3 is implicated in PHAII type E (4, 33). Activation of WNK1 stimulates SGK1 (serum- and glucocorticoid-inducible protein kinase 1), which activates the NCC, increasing sodium reabsorption, and leading to hypertension (34).

*Epidemiology*: Hypertension generally manifests in adolescence to adulthood, but cases involving younger patients have been reported (31). It is important to note that Spitzer-Weinstein syndrome in children has similar clinical features, such as metabolic acidosis and hyperkalemia, and is thought to be an early presentation of Gordon syndrome (13). The prevalence of this disorder is unknown; only about 180 cases have been reported (31).

*Workup:* No criteria for the diagnosis of PHAII has been officially published. A clinical history and family history of a first-degree relative would aid in diagnosis, but a first degree-relative is not necessary. Molecular genetic testing is ideal for diagnosis, due to variation in severity and presentation.

*Treatment:* Electrolyte and blood pressure abnormalities can be treated with a low dose thiazide, which directly inhibits NCC hyperactivity. Patients should have routine follow up for assessment of electrolyte levels and blood pressure (31). Counseling patients to adhere to a low-sodium diet is an important consideration in long-term management of hypertension.

## High Aldosterone Monogenic Hypertension

Case 6: A 15-year-old boy presented to the emergency room with projectile vomiting, migraine and hypertension. The patient had no significant past medical or surgical history. A CT scan of the head showed that the patient had a minor bleed. A cerebral angiogram showed rupture of a small aneurysm. Appropriate management, including acute blood pressure reduction, was performed and the patient survived. He presented to the clinic for a follow up visit. He is asymptomatic but still has elevated blood pressure, and laboratory testing demonstrates hypokalemia. All other labs are normal, except for low renin and elevated aldosterone levels. Adrenal vein sampling shows elevated levels of ACTH, and the patient is diagnosed with Familial Hyperaldosteronism type 1. The patient was started on dexamethasone and potassium supplements. Follow-up shows resolution of the hypertension.

### Familial Hyperaldosteronism Type 1 (Glucocorticoid-Remediable Aldosteronism)

**Figure 6:**
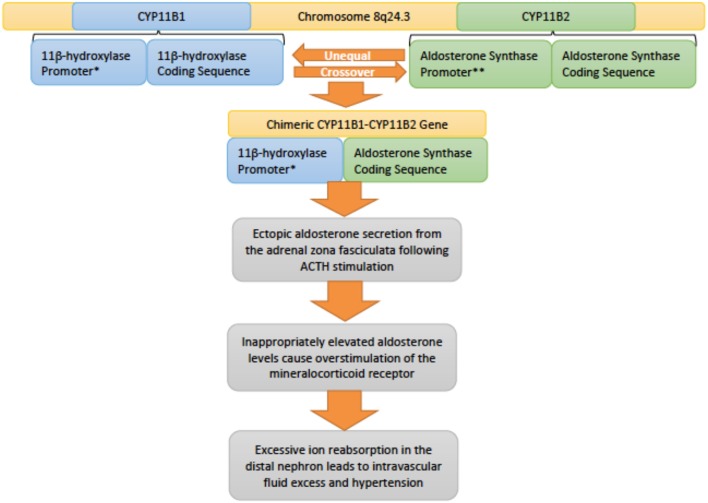
An overview of the genetic pathophysiology of familial hyperaldosteronism type I. ^*^ Promoter responsive to adrenocotropic hormone (ACTH). ^**^Promoter responsive to angiotensin II.

*Genetics and pathophysiology:* Inherited in an autosomal dominant fashion, familial hyperaldosteronism type 1 (FHT-I) is also also known as glucocorticoid-remediable aldosteronism (GRA) (20). This condition is due to alterations in the CYP11B1 (11β-hydroxylase) and CYP11B2 (aldosterone synthase) genes that lie adjacent to each other on chromosome 8q24.3 (13, 35). Unequal crossover between these genes results in a chimeric variant that incorporates regulatory elements of CYP11B1 with the coding sequence of CYP11B2 (Figure 6). As a result the gene is regulated by ACTH rather than angiotensin II, however, the gene still controls aldosterone secretion (36). Normally, aldosterone is secreted by the zona glomerulosa of the adrenal gland, but due to this chimeric gene, aldosterone is ectopically secreted from the adrenal zona fasciculata instead (37). Increased production of aldosterone drives the key clinical features of hypertension and hypokalemia in the setting of suppressed renin levels (20).

*Epidemiology:* Most patients with this disease present with severe hypertension in childhood or even infancy. These patients also suffer from a high incidence of intracerebral hemorrhage (11–18%) with a high associated mortality rate (61%) at an early age (31.7 ± 11.3 years) (38).

*Workup:* Brain imaging with computed tomography angiogram (CTA) or magnetic resonance angiography (MRA) may be warranted due to clinical presentation and a strong family history of cerebrovascular incidents and early onset of hypertension (39, 40). Due to high rates of cerebrovascular events in GRA patients, screening is recommended every 5 years after puberty (41). Hypokalemia is usually mild and few go on to develop metabolic alkalosis. The plasma aldosterone (ng/dL) to plasma renin activity (ng/mL/h) ratio usually > 30 (normal <20). Many other diagnostic modalities such as the dexamethasone suppression test, adrenal imaging, and adrenal vein sampling may help in distinguishing FHT-I from other forms of hyperaldosteronism (13).

*Treatment:* Treatment for FHT-I includes glucocorticoids which suppress ACTH, and thus prevent the secretion of aldosterone (36). If glucocorticoids do not reduce the blood pressure, then mineralocorticoid receptor antagonists, such as spironolactone, or sodium epithelium receptor antagonists, such as triamterene or amiloride, can be used as second line therapy (39).

**Case 7:** A 14-year-old female presented with back pain and muscle spasms. The patient has been complaining of muscle spasms for the past 2 months but she believed it was due to her menstrual cycle. She was initially sent to obstetrics/gynecology for exploratory laparoscopy to rule out endometriosis. The exploratory laparoscopy was negative for endometriosis but the patient was noted to be hypertensive. She has a family history of hypertension. Laboratory work up was benign. A CT scan was completed and an adrenal tumor was identified. Biopsy revealed that it was a benign adenoma. Plasma aldosterone (ng/dL) to plasma renin (ng/mL/h) ratio was well above normal (normal <20). The patient was diagnosed with familial hyperaldosteronism type 1. The patient was given glucocorticoids and asked to come back the following week. The following week, the patient still had the same complaints and elevated blood pressures. The patient was then given mineralocorticoid receptor antagonists which lead to resolution of her symptoms and hypertension. The patient's diagnosis was changed from familial hyperaldosteronism Type 1 to Type 2.

### Familial Hyperaldosteronism Type 2

Familial Hyperaldosteronism type 2 (FHT-II) is similar to FHT-I in that ectopic aldosterone synthesis occurs due to the loss of negative feedback seen with physiologic aldosterone secretion (42). However, the aldosterone synthase gene is not controlled by ACTH as in FHT-I, causing a non-dexamethasone-suppressible form of hyperaldosteronism. FHT-II had previously been mapped to chromosome 7p22, but no specific gene at this locus has been identified as the cause of the disease (43, 44). A recent study of patients diagnosed with FHT-II implicates gain-of-function mutations in the CLCN2 gene, encoding a chloride channel, ClC-2, located at chromosome 3q27.1 (45). The variant channels were shown *in vitro* to cause depolarization of adrenocortical cancer cell lines, leading to increased expression of aldosterone synthase and aldosterone production (45). FHT-II is characterized by bilateral adrenocortical disease and adenomas (42). The diagnosis of FHT-II should be confirmed by positive family history because FHT-II is clinically and biochemically indistinguishable from non-inherited primary aldosteronism (13). Genetic testing for CLCN2 mutations may prove helpful, though further work is needed to identify other possible causative genes (45). The mainstay of treatment for this disease is mineralocorticoid receptor antagonists or unilateral adrenalectomy (4).

### Familial Hyperaldosteronism Type 3

Familial hyperaldosteronism type 3 (FHT-III) is an autosomal dominant condition caused by gain-of-function mutations in the KCNJ5 gene located at locus 11q24.3. KCNJ5 encodes a potassium channel, which loses its ionic selectivity in disease-causing variants to allow other cations, particularly sodium, to pass through. This allows for depolarization of the adrenal cortical cell, enhancing the expression of aldosterone synthase and subsequently raising aldosterone levels in patients (44). Treatment is similar to FHT-II and primary aldosteronism, ranging from mineralocorticoid antagonists to possible adrenalectomy depending on the severity of the hypertension (44, 46).

### Familial Hyperaldosteronism Type 4

Familial hyperaldosteronism type 4 (FHT-IV) is an autosomal dominant condition caused by gain-of-function mutations in the CACNA1H gene located at chromosome 16p13.3 (44). CACNA1H encodes a transient opening calcium channel localized to the zona glomerulosa. The mutations implicated in FHT-IV cause the channel to be more likely to open at the baseline electrochemical gradient and remain open longer upon activation (47). These changes allow for greater influx of calcium ions, stimulating adrenal cortical cells and subsequently activating aldosterone synthase (47). As with other FHT types, treatment depends on the severity of the condition, possibly varying on the specific mutation, and ranges from medical management with mineralocorticoid antagonists to potential surgical adrenalectomy (46).

## Monogenic Hypertension due to Adrenergic/Sympathetic Excess

**Case 8:** A 7-year-old boy was brought to the clinic with a pounding headache. The child had also complained of palpitations for the past year. The patient had no other complaints and had been healthy since birth. The patient was noted to have a severely elevated blood pressure of 210 / 130 mmHg. Physical exam was otherwise normal and routine laboratory values showed no significant findings. Urine metanepharines were collected and found to be elevated. Abdominal CT scan demonstrated a tumor adjacent to the adrenal glands. The diagnosis of pheochromocytoma was made and the tumor was surgically removed after appropriate perioperative blockade.

### Familial Pheochromocytoma

*Genetics and pathophysiology:* Familial pheochromocytoma describes increased susceptibility to pheochromocytoma due to a variety of well-documented genetic mutations. Pheochromocytoma typically presents with paroxysmal symptoms, such as episodes of severe hypertension. Release of norepinephrine and epinephrine, parituclarly the former, is above and beyond physiologic levels. Pheochromocytoma susceptibility can be inherited alone or as a part of several syndromes. Von Hippel-Lindau disease (VHL) is associated with bilateral pheochromocytomas, retinal and cerebellar angiomas, renal and pancreatic cysts, and renal cell carcinoma. The mutation is on chromosome 3p25.3 and is a tumor-suppressor gene defect that leads to disease (48). RET, a proto-oncogene, is associated with type 2 multiple endocrine neoplasia syndrome (MEN 2) as well as non-syndromic pheochromocytoma (49). MEN 2 is inherited in autosomal dominant fashion due to a mutation on chromosome 10q11.21. MEN 2 has two subtypes MEN 2A and 2B. MEN 2A is associated with pheochromocytoma, medullary carcinoma of the thyroid, and hyperparathyroidism while MEN 2B is associated with pheochromocytoma, medullary carcinoma of the thyroid, and mucosal neuromas (50). Pheochromocytoma has also been associated with neurofibromatosis type I, caused by mutations in the NF1 gene, located at chromosome 17q11.2 (51). Solitary pheochromocytomas have also been shown to contain mutations in the aforementioned genes. One study of solitary tumors reported that 86% contained copy number alterations in genes associated with familial pheochromocytoma; changes in NF1 were found to be the most frequent at 26% of the tumors studied (52).

*Epidemiology:* Initially pheochromocytomas were considered to have 10% familial and 90% sporadic development. New technology in genetic testing has demonstrated that pheochromocytomas are 50% sporadic with 15–25% due to germ line mutations (53).

*Workup:* Catecholamine metabolism studies such as plasma free metanephrines and urinary fractionated metanephrines allow for successful screening of the condition and can be monitored for treatment response (54).

*Treatment:* Per The Endocrine Society clinical practice guideline, the treatment of functional pheochromocytomas begins with initiation of antihypertensive therapy followed by tumor resection. Medical management prior to surgery, known as pre-operative blockade, utilizes alpha-adrenergic antagonists (e.g., phenoxybenzamine or doxazosin) as firstline therapeutics. Other antihypertensives, particularly dihydropyridines and beta-adrenergic antagonists, may be used supplementarily. Diligent monitoring of blood pressure and catecholamine metabolism should take place throughout the perioperative course. Due to pheochromocytoma's association with the various aforementioned neoplastic syndromes, genetic testing may be recommended as a prognostic and preventative indicator (54).

## Monogenic Hypertension due to Vascular Smooth Muscle Proliferation

### Hypertension and Brachydactyly Syndrome

Hypertension and brachydactyly (HTNB) describes an autosomal dominant syndrome caused by a mutation in the PDE3A gene located at chromosome 12p12.2; this gene encodes a phosphodiesterase that hydrolyzes cAMP. Variants of PDE3A that cause HTNB exhibit a gain-of-function due to altered enzyme phosphorylation (21). The mutant PDE3A enzymes consequently decrease cellular cAMP levels in vascular smooth muscle cells, allowing for proliferation. Uncontrolled proliferation of the smooth muscle eventually narrows the lumen of vessels, raising blood pressure. The associated brachydactly also stems from decreased cAMP levels, which lower the levels of PTHrP (parathyroid hormone related protein), a key moderator of chondrogenesis. Treatment aims to restore normal cAMP levels with high concentration milrinone, and there is possible benefit to increasing cGMP levels with phosphodiesterase inhibitors (21).

## Conclusion

Hypertension is known as the silent killer; if not managed properly, it can lead to progressive end organ damage and death. Essential hypertension is often the result of multiple patient factors including polygenic inheritance and environmental factors, such as diet and physical activity. Monogenic hypertension refers to specific genetic mutations that interfere with normal renal and adrenal regulation of blood pressure. All forms of monogenic hypertension have low renin and can be further classified by the levels of aldosterone. Accurate diagnosis may require further hormonal studies or genetic testing. It important to have a high level of clinical suspicion and precisely diagnose these patients, as treatment is often quite different from traditional patients with essential hypertension. Appropriate treatment can decrease the morbidity and mortality associated with uncontrolled hypertension. With future advancements in genetic testing modalities and treatment options, there is hope for earlier recognition and improved management of the various forms of monogenic hypertension.

## Author Contributions

All the authors are responsible for the literature review, drafting and revision of the manuscript, and approved the final version of the manuscript.

### Conflict of Interest Statement

The authors declare that the research was conducted in the absence of any commercial or financial relationships that could be construed as a potential conflict of interest.
